# The Vallecas Project: A Cohort to Identify Early Markers and Mechanisms of Alzheimer’s Disease

**DOI:** 10.3389/fnagi.2015.00181

**Published:** 2015-09-30

**Authors:** Javier Olazarán, Meritxell Valentí, Belén Frades, María Ascensión Zea-Sevilla, Marina Ávila-Villanueva, Miguel Ángel Fernández-Blázquez, Miguel Calero, José Luis Dobato, Juan Antonio Hernández-Tamames, Beatriz León-Salas, Luis Agüera-Ortiz, Jorge López-Álvarez, Pedro Larrañaga, Concha Bielza, Juan Álvarez-Linera, Pablo Martínez-Martín

**Affiliations:** ^1^Gregorio Marañón University Hospital, Madrid, Spain; ^2^Alzheimer’s Center Reina Sofía Foundation – CIEN Foundation and CIBERNED, Carlos III Institute of Health, Madrid, Spain; ^3^Laboratory of Medical Imaging Analysis and Biometrics, Rey Juan Carlos University, Móstoles, Spain; ^4^Department of Artificial Intelligence, Technical University of Madrid, Boadilla del Monte, Spain; ^5^Department of Neuroimaging, Hospital Ruber Internacional, Madrid, Spain; ^6^National Center of Epidemiology and CIBERNED, Carlos III Institute of Health, Madrid, Spain

**Keywords:** Alzheimer’s disease, cohort study, early detection, mild cognitive impairment, risk factors

## Abstract

**Introduction:**

Alzheimer’s disease (AD) is a major threat for the well-being of an increasingly aged world population. The physiopathological mechanisms of late-onset AD are multiple, possibly heterogeneous, and not well understood. Different combinations of variables from several domains (i.e., clinical, neuropsychological, structural, and biochemical markers) may predict dementia conversion, according to distinct physiopathological pathways, in different groups of subjects.

**Methods:**

We launched the Vallecas Project (VP), a cohort study of non-demented people aged 70–85, to characterize the social, clinical, neuropsychological, structural, and biochemical underpinnings of AD inception. Given the exploratory nature of the VP, multidimensional and machine learning techniques will be applied, in addition to the traditional multivariate statistical methods.

**Results:**

A total of 1169 subjects were recruited between October 2011 and December 2013. Mean age was 74.4 years (SD 3.9), 63.5% of the subjects were women, and 17.9% of the subjects were carriers of at least one ε4 allele of the apolipoprotein E gene. Cognitive diagnoses at inclusion were as follows: normal cognition 93.0% and mild cognitive impairment (MCI) 7.0% (3.1% amnestic MCI, 0.1% non-amnestic MCI, 3.8% mixed MCI). Blood samples were obtained and stored for future determinations in 99.9% of the subjects and 3T magnetic resonance imaging study was conducted in 89.9% of the volunteers. The cohort is being followed up annually for 4 years after the baseline.

**Conclusion:**

We have established a valuable homogeneous single-center cohort which, by identifying groups of variables associated with high risk of MCI or dementia conversion, should help to clarify the early physiopathological mechanisms of AD and should provide avenues for prompt diagnosis and AD prevention.

## Introduction

Alzheimer’s disease (AD) is a devastating illness affecting more than 25 million people worldwide (Prince et al., 2013) and producing a tremendous personal and societal impact (Wimo et al., 2013). Despite the important amount of resources conveyed for drug research, advances in the treatment of AD have been scarce, which underlines the complexity of the disease and the need of a better understanding of its etiology and pathogenesis leading from healthy state to full-blown dementia. The relative failure of the different therapy approaches has reinforced the search for alternative ways of fighting the disease, particularly prevention (Smith and Yaffe, 2014). A better understanding of the risk factors, especially those modifiable, and of the earliest biological and clinical signs of the disease, is crucial in order to implement effective preventative strategies and future therapies.

Current knowledge understands AD as a continuum process starting many years before the onset of clearly noticeable symptoms (Bateman et al., 2012). A high degree of agreement has been reached around the different stages of the disease, namely the preclinical phase (brain changes without symptoms), a prodromal mild cognitive impairment (MCI) phase, and a final dementia phase usually lasting 10–15 years, leading to total dependence and, eventually, patient’s death (Dubois et al., 2010; Albert et al., 2011; Sperling et al., 2011). In practical terms, of utmost interest is the recognition of the clinical and biological characteristics of people that converts from normality to MCI and from MCI to dementia, as well as the factors that may accelerate or prevent those transits.

The passage from normality to early, mild signs of cognitive impairment is difficult to categorize and isolate. A potential feature appearing during this passage is the so-called “subjective cognitive impairment” (SCI) (Reisberg and Gauthier, 2008), or “subjective cognitive decline” state (Jessen et al., 2014), with prevalence rates in old people varying from 10 to 60%, depending on age, setting, and definitions applied (Garcia-Ptacek et al., 2013). Possibly due to the lack of a precise definition, studies addressing the conversion from normal cognition (NC) to MCI have been scarce, with incidence rates varying between 51 and 77 per 1000 person-years. The most frequently reported risk factors for incident MCI are higher age, lower education, and hypertension (HTA) (Luck et al., 2010). Conversion from MCI to dementia has been more widely studied, with a duration of 7–10 years for the MCI stage and annualized conversion rates of 8–17 per 100 person-years (Ward et al., 2013). Cognitive performance, cortical amyloid deposition, hippocampal atrophy, hypometabolism in the parietotemporal cortex, and alteration in the cerebrospinal fluid (CSF) levels of 42-aminoacid amyloid beta peptide (Aβ_42_), tau, and phosphorylated tau (p-tau) proteins have been consistently associated with higher conversion rates from MCI to AD dementia (Brooks and Loewenstein, 2010; Heister et al., 2011; Barnes et al., 2014). Other markers or comorbidities (e.g., vascular factors, sleep disturbance) may also be of relevance in the transitions from healthy state to AD (Dufouil et al., 2005; Frisardi et al., 2010; Osorio et al., 2011).

Clearly, the targets for early AD identification should be the states of SCI and MCI, along with the characterization of those subjects with high risk of conversion to dementia. Under a multicausal model of aging-associated, late-onset, sporadic AD, multiple markers are expected to be relevant for the detection of the groups of interest and, therefore, the most cost-effective and safest procedures should be prioritized. Cognitive and magnetic resonance imaging (MRI) measures are useful for detecting and characterizing subjects with SCI and MCI, as well as for identifying those subjects who will develop dementia (Dickerson et al., 2013) but, in the case of MRI, the cost is high. Determinations of brain amyloid deposits or CSF markers (Aβ42, tau, and p-tau proteins) are highly predictive of AD conversion in subjects with MCI, but the procedures are either expensive [positron emission tomography (PET)] or uncomfortable (CSF determination). Blood-based (or peripheral) biomarkers (e.g., Aβ fractions, oxidative stress, and inflammatory markers) are particularly attractive in late-onset AD because they can be comfortably and inexpensively retrieved. In the last years, promising data have been published regarding the potential of peripheral biomarkers for the early diagnosis of AD (Carmona et al., 2013; Mapstone et al., 2014).

We launched a single-center, longitudinal, cohort study with yearly evaluations to identify subjects at the initial stages of AD and to clinically and biologically characterize the transitions between healthy cognition, SCI, MCI, and AD dementia. The study is focused on cognitive testing, comorbidities (particularly vascular factors, sleep disturbance, and medications), multi-modal MRI, and systematic blood collection. The objective is twofold: first, to identify a high risk profile for developing cognitive impairment and AD in cognitively healthy old people and, second, to shed light on the multiple mechanisms that may lead from healthy cognition to dementia in that segment of the population. This strategy should facilitate, in the mid-term, the testing of disease modifying therapies and, next, the treatment of the populations at risk, before they become cognitively impaired or demented.

## Materials and Methods

### Design

Single-center, observational, 4-year longitudinal standardized study, with yearly assessments.

### Subjects

Volunteers were recruited through radio and TV campaigns, leaflet distribution, and visits of the research team to social centers for the elderly.

The inclusion criteria were as follows: (1) community-dwelling individuals; (2) both sexes; (3) from 70 to 85 years of age; (4) able to manage and independent life without any mental disorder (cognitive or psychiatric) impeding daily functioning; (5) with reasonable expectation of survival at a 4-year period, operationalized as absence of any severe disease at recruitment; and (6) signed informed consent.

The exclusion criteria were as follows: (1) dementia or severe cognitive deterioration, operationalized as Mini Mental Statement Examination (MMSE) (Folstein et al., 1975) below 24 and functional activities questionnaire (FAQ) (Pfeffer et al., 1982) scores below 6 at the baseline assessment; (2) history of neurological disease with clinically relevant impact on cognition (e.g., cerebrovascular disease); (3) severe psychiatric disorder; (4) incidental structural brain findings with impact on cognitive impairment or survival (e.g., malignant brain tumor); (5) presence of a severe systemic disease (e.g., cancer under treatment, malignant hypertension, etc.); and (6) problems for understanding spoken or written Spanish language.

It was anticipated that two population groups would be represented in the cohort: (1) subjects without noticeable risk factors for dementia or AD and (2) subjects with risk factors for dementia or AD. The following factors were considered: subject or informant report of cognitive deterioration, MCI, AD in first-degree relative, vascular risk factors (VRF) (high blood pressure, ischemic heart disease, atrial fibrillation, diabetes, dyslipidemia, obesity, smoking, stroke), and low education level, intellectual activity, or socioeconomic class. The presence of any of the four first factors or a combination of at least two of the other factors was required to consider a participant as carrier of risk for dementia or AD. The assumption was that the sample would be evenly distributed for each group.

### Sample Size

Age interval of 70–85 years was chosen aiming at a balance between high incidence of dementia and survival at a 4-year follow-up horizon. Under 70 years of age, the incidence of dementia is around 0.6% per year (Hebert et al., 1995), hence requiring the recruitment of a considerable number of cognitively healthy participants for observing a significant number of MCI or dementia conversions. At the opposite end of the study target age, the risk of mortality for the population between 85 and 90 years of age increased in average around 15 per 1000 per year (Spanish National Institute of Statistics estimations for 2008) (National Institute of Statistics, 2014), which would cause a considerable attrition of the sample.

At initial setup of the study, population aged 70–85 was around 4,845,000 persons in Spain, with the following distribution: 70–74 years, 38.4%; 75–79 years, 34.5%; 80–85 years, 24.4%; and 85 years, 2.7%. Assuming a similar distribution for the study population and considering the incidence figures from previous investigations (Hebert et al., 1995; McDowell, 2001; Kukull et al., 2002; Bermejo-Pareja et al., 2008a) it was calculated that 20–21 new cases of dementia, including 13–15 cases of AD, would be diagnosed per 1000 person-years. These figures should increase over time, as the cohort becomes older during the 4-year follow-up, and also considering the conversion of a proportion of those participants who will present MCI at baseline. Since attrition of the sample was calculated as around 5% per year, a sample of 1200 participants was estimated to observe 100 cases of incident dementia, including 75 cases of AD at the fourth year of follow-up. For that sample size and a foreseen incidence of MCI of 2–4% in the 70-to 85-year-old population (Mielke et al., 2014), conversion from NC to MCI is expected to occur in over 150 subjects. These figures were considered sufficient for the study of the potential markers and risk factors.

### Assessments

**Figure 1:**
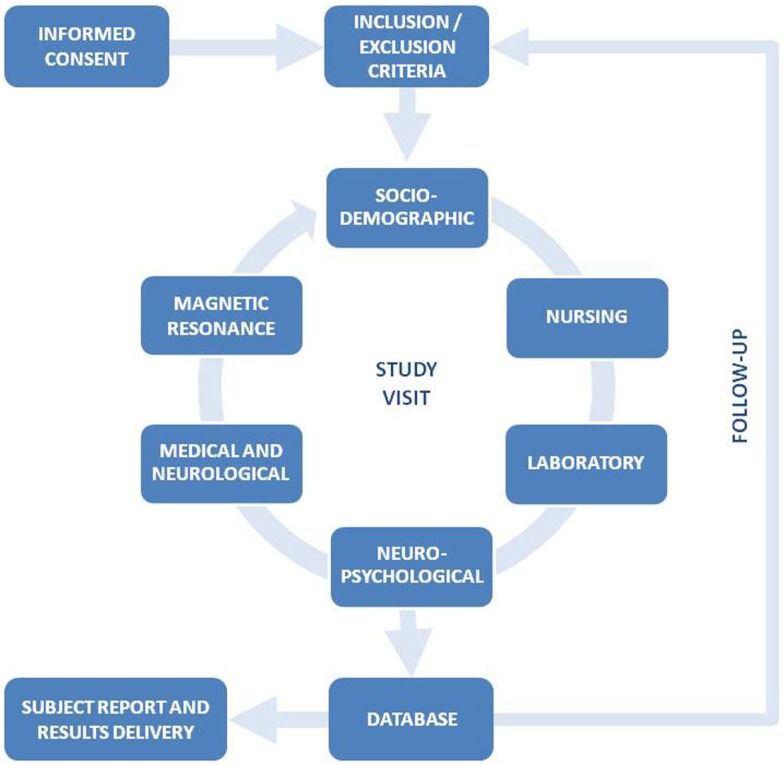
**General procedure and different parts of the study visit of the Vallecas Project**.

The general procedure of the Vallecas Project (VP) is shown in Figure 1. After participants consent, inclusion and exclusion criteria are checked and the baseline assessment visit is conducted. Sociodemographic data, vital signs, and blood samples are collected first, followed by neuropsychological, medical, and MRI assessment. The complete study visit is usually carried out in a single day, with convenient breaks. The total duration for the study visit is 4 h.

#### Sociodemographic Assessment

Subject demographic and family data, as well as data regarding lifestyle, subjective well-being, and quality of life (QoL) are completed by the participants themselves, with assistance from the investigators when needed. The sociodemographic variables were elaborated *ad hoc*, after reviewing the main epidemiological studies regarding lifestyle and AD (Table 1).

**Table 1:** **Sociodemographic, clinical, and neuropsychological variables collected in the study visit**.

	Variable group	Variable	VISIT^a^				
			1	2	3	4	5
Sociodemographic	Demographic data	Race, sex, marital status, number of children, living situation, socioeconomic status, place of residence, occupation, education, number of siblings, ages of parents when the subject was born					
	Lifestyle	Eating and sleep habits, social relationships and free time, physical activity, expectations, beliefs					
	Quality of life and subjective well-being	Movement, self-care, routines, pain/discomfort, anxiety/depression, past and current subjective well-being					
Nursing	Vital signs and morphology	Blood pressure (seated and standing), height and weight measures, head and waist circumference					
Medical and neurological	Medical interview	Educational achievement and handedness					
		Vascular risk factors and vascular diseases (present or past): hypertension, hypotension, diabetes, carbohydrate intolerance, hypercholesterolemia, hypertriglyceridemia, smoking, overweight/obesity, myocardial infarction/angina, atrial fibrillation, other cardiac diseases, stroke or transient ischemic attack, Hachinski scale					
		Secondary causes of cognitive deficit (present or past): alcohol, drugs, toxics and nutritional deficits, mental health problems (including anxiety and depression), head injury, neurological diseases (cerebrovascular episodes, headache, movement disorder, epilepsy, infection, inflammation, tumors, neuromuscular disorders, toxic and deficiency diseases, development delay, pain)					
		General medical and surgical anamnesis, particularly looking for disorders that could produce cognitive dysfunction (thyroid disorders, hepatic failure, renal failure, obstructive sleep apnea syndrome, etc.)					
		Sleep habits and disorders					
		Reproductive history (women)					
		Current treatments (pharmacological and non-pharmacological)					
		Vision and hearing problems					
		Cognitive symptoms (attention, orientation to place, immediate memory, delayed memory, visual recognition, dysphasia, executive dysfunction, dyspraxia)					
		Neuropsychiatric symptoms (hallucinations, delusions, agitation, depression, anxiety, irritability, disinhibition, euphoria, apathy, aberrant motor behavior, sleep and night-time behavior change, appetite, eating change)					
		Physical symptoms (falls, tremor, loss of consciousness, gait abnormality, urinary incontinence, seizures, focal neurological symptoms)					
	Family history	Number and current age (or age of the death) of first-degree relatives and age at onset of neurological or psychiatric illness					
	Medical examination	Heart auscultation					
		Neurological examination (cranial nerves, motor system, sensory system, osteotendinous and primitive reflexes, cerebellum, gait)					
		Timed “up-and-go” test and finger tapping test					
Neuropsychological	Cognitive performance	Reading test for estimation of intelligence					
		Mini mental state examination					
		Free and cued selective reminding test					
		Clock drawing test					
		Fonetic verbal fluency (P, M, R)					
		Semantic verbal fluency (animals, fruits and vegetables, kitchen tools)					
		Digit-symbol coding of the wechsler adult intelligence scale (WAIS-III)					
		Rey–Osterrieth complex figure test					
		Digit span forward and backward of the WAIS-III					
		Symbolic gesture and bilateral imitation of postures of the revised Barcelona test					
		Rule shift cards of the behavioral assessment of dysexecutive syndrome					
		Five point test					
		Boston naming test (15-item version)					
	Subjective memory complaints	Memory complaints scale (*ad hoc*)					
		Memory failures in everyday					
	Depression and anxiety	Geriatric depression scale (15-item version)					
		State-trait anxiety inventory					
	Functional scales	Functional activities questionnaire					
		Clinical dementia rating					

*^a^Gray cells indicate that the variables were collected at the corresponding study visit, whereas white cells indicate that the variables were not collected at that visit*.

#### Vital Signs and Morphometry

Blood pressure (seated and standing), height and weight, and head and waist circumferences are measured by a nurse during all the study visits.

#### Medical and Neurological Assessment

A semi-structured medical interview, focused on VRF, neurological disorders, psychiatric disorders, current medications, family history of dementia, and sleep habits is conducted by a neurologist, followed by a medical and neurological exam, which includes some brief motor tasks (Podsiadlo and Richardson, 1991; Ashendorf et al., 2009) (Table 1).

#### Neuropsychological Assessment

It includes a comprehensive neuropsychological battery (Table 1) designed to assess the following cognitive domains, which are relevant for MCI or dementia diagnosis: processing speed (Wechsler, 1997) visual and verbal episodic memory (Rey, 1941; Osterrieth, 1944; Buschke, 1984; Peña-Casanova et al., 2009a), executive functions (Regard et al., 1982; Lee et al., 1994; Peña-Casanova, 2005), language (Peña-Casanova et al., 2009b), and visuospatial and visuoconstructive skills (Rey, 1941; Osterrieth, 1944; Lee et al., 1994; Cacho et al., 1999). The battery is also expected to be sensible for the detection of future cognitive changes in the specific cognitive domains. In addition, scales of subjective memory complaints (Sunderland et al., 1984), mood (Yesavage et al., 1982–1983), anxiety (Spielberger et al., 1970), and informant-based functional scales (Pfeffer et al., 1982; Morris, 1993) are administered. The neuropsychological battery is administered by neuropsychologists.

#### Laboratory

In order to maximize the chances for biomarker discovery, three types of evacuated blood collection tubes for serum, plasma, and blood cells are obtained at each study visit by trained technologists using a butterfly connected to a vacuum tube holder. In all cases, blood samples are processed within 1 h of procurement by standard procedures. From these procedures, eight different fractions, namely whole blood, serum, platelet-rich plasma, platelet-poor plasma, buffy coat, red blood cells, mononuclear blood cells, and genomic DNA are obtained and kept in duplicate aliquots at −80°C. Genotyping of apolipoprotein E (*APOE*) polymorphisms (rs429358 and rs7412) is performed by real-time PCR (Calero et al., 2009). Additionally, in order to define homogenous groups and refine performance of biomarkers, several polymorphisms clearly associated to AD are studied by using TaqMan^®^ probes, namely, *BIN1* (rs744373), *CLU* (rs11136000), *ABCA7* (rs3764650), *CR1* (rs3818361), and *PICALM* (rs3851179).

#### Neuroimaging

All studies are carried out in a 3-T MRI (Signa HDxt GEHC, Waukesha, WI, USA) equipped with a gradient system of 50 mT/m. A phased array eight channels brain coil is used for all the subjects. The VP protocol includes a structural study with T1 sequences for volumetry and FLAIR and T2* sequences to assess white matter (WM) lesions and microhemorrages. Perfusion study with arterial spin labeling (ASL) technique is conducted to check for functional alterations and diffusion tensor imaging (DTI) study is performed for analysis of the anisotropy of WM. In addition, the DTI sequence permits to measure structural connectivity. Finally, the VP neuroimaging protocol includes a resting state functional study with blood oxygen level dependent (BOLD) sequences (rs-fMRI) to analyze functional connectivity.

Magnetic resonance imaging acquisition parameters are as follows:

– 3DT1 sequence: 3D FSPGR with IR preparation (TR 10 ms, TE 4.5 ms, TI 600 ms). FOV 240 mm, matrix 288 × 288, slice thickness 1 mm.
– FLAIR: axial 2D FSE IR (TR 9000 ms, TE 130 ms, TI 2100 ms). FOV 24 mm, slice thickness 3.4 cm.
– T2*: axial 2D GRE EPI (TR 3475 ms, TE 27 ms). FOV 240 mm, matrix 262 × 192, slice thickness 3 mm.
– Perfusion: 3D ASL acquisition with a delay of 2025 ms. FOV 240 mm, slice thickness 4 cm.
– DTI: single shot DWI SE-EPI (TR 9200 ms, TE 80 ms) with a-value of 800 s/mm^2^ and 21 gradient directions. FOV 240 mm, matrix 128 × 128, slice thickness 3 mm.
– Rs-fMRI: acquisition without task, 5:10 s (TR 2500 ms, TE 27 ms). FOV 240 mm, matrix 96 × 96, slice thickness 2.6 cm.

### Cognitive diagnoses at baseline

After the study visit is completed, one of the following cognitive diagnoses is given by consensus of neurologist and neuropsychologist.

– NC. Performance in neuropsychological tests is considered within the expected range (not inferior to 1.5 SD or 5° percentile) for the participant age and education, with or without cognitive complaints.
– MCI. There are cognitive complaints by participant or informant, performance on cognitive tests falls below what is expected (below 1.5 SD or 5° percentile) according to participant age and education, and usual activities of daily living (ADL) are essentially preserved (Winblad et al., 2004). In case of MCI diagnosis, a type of amnestic (aMCI), non-amnestic (naMCI), or mixed (i.e., amnestic and non-amnestic) (mMCI) MCI is further defined, according the participant’s neuropsychological performance.
– Dementia. The diagnosis of dementia is established according to the fourth edition, text revised, of the Diagnostic and Statistical Manual of Mental Disorders (DSM-IV-TR) (American Psychiatric Association (APA), 2000). Any patient with dementia diagnosis at the baseline visit is excluded from the study.

### Follow-up visits

Participants of the VP are expected to complete one baseline and four follow-up annual assessment visits. Subjects are contacted by telephone to arrange the next study visit. The above described baseline assessment is essentially repeated yearly, with minor modifications as follows: (1) lifestyle questionnaire from the sociodemographic assessment is not administered at the three intermediate visits and medical interview is also shortened, focusing on changes or new medical events since the last assessment (particularly, new medical conditions, medications, and change in cognitive symptoms) and (2) the neuropsychological battery is mildly modified at the follow-up assessments: the Rey–Osterrieth Complex Figure Test is not administered at two of the intermediate visits and some new tests focusing on language (Kaplan et al., 1983; Goodglass and Kaplan, 1996; Fernández-Blázquez et al., 2012), executive (Regard et al., 1982), and visuoespatial functions (Lee et al., 1994), which may contribute to a more precise diagnosis of MCI or dementia or to research purposes, are included. A cognitive diagnosis is conducted after each follow-up visit, using the methods described for the baseline visit.

If a participant cannot attend a follow-up visit, he/she is invited to perform a medical interview by phone. This interview is composed of questions regarding new medical conditions or events, current medications, cognitive symptoms, and a brief mental status exam (i.e., temporal orientation, memory, and calculation items of the MMSE), as well as interview with an informant, performance of FAQ, and diagnosis of dementia according to DSM-IV-TR (American Psychiatric Association (APA), 2000). Finally, the participant is encouraged to remain into the study and return next year for assessment.

### Feedback for participants

Formal feedback is provided in the form of written reports of the study visit that participants receive at home. These reports include general results of neuropsychological tests and MRI study and, if clinically relevant, results from the nursing and medical and neurological assessments. Furthermore, if any procedure of the VP reveals findings requiring medical or psychiatric attention, the subject is referred to the appropriate assistance resource or is contacted by telephone to provide information about the finding and the steps to follow.

### Data analysis

In addition to descriptive statistics, such as central tendency and distribution methods, the comparisons will be analyzed with the Student’s *t*-test for paired and unpaired populations, ANOVA for one factor, and ANOVA for repeated measures or Wilcoxon, Mann–Whitney, and Kruskal–Wallis tests if variables do not meet assumptions for the use of parametric methods. Analyses of association will be carried out by means of correlation coefficients (Pearson, Spearman, Kendall) and linear and logistic regression. Predictors will be explored with multiple regression models. Survival analysis and Cox proportional hazards models for time unit, considering the conversion to dementia as the event of interest, will be built. Logistic regression models controlled by age (as a linear variable) and sex will be used to calculate adjusted odds ratio (OR) and 95% confidence intervals (CI) for the different variables of interest (i.e., potential predictors of MCI or dementia conversion, e.g., *APOE*, VRF, MRI variables, etc.). Deviations from normality of quantitative variables will be checked by the Kolmogorov–Smirnov statistic with Lilliefors’ significance. The statistical packages IBM SPSS Statistics (IBM Corp., Armonk, NY, USA), R (R Foundation for Statistical Computing, Vienna, Austria), and Stata (StataCorp LP, College Station, TX, USA) will be used.

Given the exploratory nature of the VP, multidimensional statistics and machine learning methods will be also applied to the produced data. Three types of analyses will be carried out, which will allow any kind of inference (diagnostic, predictive, intercausal, and abductive):

– Longitudinal data clustering, with the aim of identifying subjects with similar behavior over time. Adaptations of partitional clustering (e.g., K-means) and probabilistic clustering (e.g., finite mixture models) for longitudinal data will be developed for covering this analysis.
– Predictive models, with the aim of predicting the time period where MCI arises and also when this evolves toward dementia. Pattern recognition techniques, such as classification trees, Bayesian classifiers, K-nearest neighbors, logistic regression, support vector machines, and ensembles of classifiers, will be adapted to the special characteristics of the data set.
– Correlation models, based on dynamic probabilistic graphical models, able to capture the dependence relationships among all types of variables (genetic, phenotypic, environmental, neuroimaging, clinical, etc.).

### Ethic aspects

The study was approved by the Ethics Committee of the Carlos III Institute of Health and the participants signed informed consent before inclusion.

## Results

**Figure 2:**
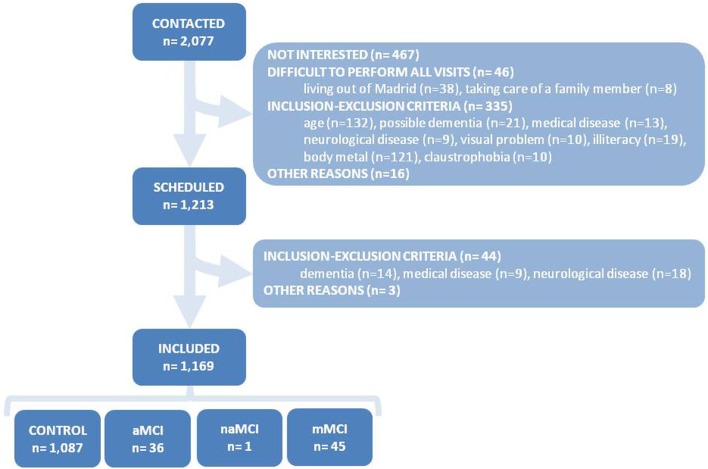
**Flowchart of subject recruitment and baseline cognitive diagnoses**. aMCI, amnestic mild cognitive impairment (MCI); naMCI, non-amnestic MCI; mMCI, mixed (i.e., amnestic and non-amnestic) MCI; NC, normal cognition.

A total of 2077 subjects contacted the study secretariat during the recruitment period (i.e., October 2011 to December 2013), but 864 of them were discarded before evaluation because they were not interested in the study or clearly met some of the study exclusion criteria (Figure 2). One of the most frequent reasons for study exclusion at that point was the presence of metallic prostheses, pacemaker, or other body metals. To circumvent that obstacle for recruitment, a paper document was designed *ad hoc*, which the volunteers had to provide, signed by the doctor who implanted the metal prosthesis, authorizing the performance of 3-T MRI study. However, that document was only provided in a minority of the cases. For that reasons, in order to accelerate the inclusion of subjects, the exclusion criteria of the VP were modified during the recruitment period, allowing the participation of subjects for whom MRI was not possible.

Of the remaining 1213 subjects, 44 subjects were discarded due to the identification of exclusion criteria during the baseline assessment. Hence, the final number of included and accepted individuals for follow-up was 1169. The cognitive diagnoses after the baseline assessment were as follows: NC 93.0%, aMCI 3.1%, naMCI 0.1%, and mMCI 3.8%. The distribution of the study subjects and the baseline cognitive diagnoses are summarized in Figure 2.

All the included subjects were of Caucasian ethnicity and virtually all of them resided in urban areas of the city of Madrid. Mean age was 74.4 (SD 3.9, range 70–85 years) and 63.5% of them were women. Concerning the educative level, 18.6% had not completed primary school, 32.8% had completed primary education, 24.2% had completed high school, and 24.4% had achieved university degree. History of dementia in first-degree relative was present in 20.8% of the subjects (1 relative, 16.7%; 2 relatives, 3.5%, and 3 relatives, 0.6%). Mother was the relative most frequently affected (progressive dementia was referred in the mother of 11.6% of the participants).

Vascular risk factors frequently reported in the study cohort. The most frequent present VRF was HTA (52.7%), followed by dyslipidemia (51.8%), diabetes mellitus (DM) (11.6%), and tobacco use (5.4%). The respective figures for a history of those conditions in the past were as follows: 1.5% (HTA), 3.0% (dyslipidemia), 2.6% (DM), and 32.2% (tobacco use). Past history of depression was also rather frequent: 22.2% of the participants referred 1 episode, 2.9% referred 2 episodes, and 6.7% referred >2 episodes of past depression. Nevertheless, a majority of participants (77.0%) perceived their health as good or very good and they rated themselves in the mid-to-high strata of socioeconomic level.

Blood samples were successfully obtained, processed, and stored from virtually all the included subjects (1168 out of 1169, 99.9%). By contrast, CSF extraction, which was offered as voluntary, was rejected by the immense majority of subjects. In fact, after information to 104 consecutive subjects, only 1 CSF sample was consented and collected. The usual reasons for spinal tap rejection were lack of interest in CSF procedure or in CSF results at that moment. For those reasons, the possibility of CSF extraction was eliminated from the VP.

The frequency of *APOE* ε4 allele was 17.9%, while the frequency of *APOE* ε2 was 10.4%. Mild decrease of frequency of the *APOE* ε4 allele was observed when the volunteers were stratified according to age (19.9% for <75 years of age, 14.5% for 75–80 years of age, and 13.3% for >80 years of age) (*p* = 0.052). There were no significant differences in the frequency of ε4 regarding sex (19.0% male, 16.9% female, *p* = 0.399), whereas the ε2 allele was more frequently observed in men than in women (13.5 vs. 9.0%, *p* = 0.022).

Baseline MRI study was successfully obtained in 1051 (89.9%) of the included participants. Reasons for not conducting the MRI study in the remaining 118 subjects were as follows: body metals (85 subjects, 72.0%), claustrophobia (19 subjects, 16.1%), incidental finding in previous MRI (12 subjects, 10.2%), and not stated reasons (2 subjects, 1.7%).

## Discussion

A single-center cohort study was launched to characterize the social, clinical, neuropsychological, and biological underpinnings of late-onset AD inception. A sample of 1171 volunteers, aged 70–85, was recruited, virtually accomplishing the initially projected sample of 1200 volunteers (97.9%). Main barriers for recruitment were lack of motivation from the potential participants and contraindication for MRI performance (Figure 2), which were circumvented by, respectively, intense work of search of candidates, information and motivation by the study administrative staff and allowance of study inclusion without MRI performance.

There was a mild predominance of women in the included subjects (63.5%), but only slightly above the prevalence of women in the population data of the Community of Madrid provided by the Spanish National Institute of Statistics (frequency of women of 58.5% according census data of July 2013) (National Institute of Statistics, 2014). However, the educational attainment of the included subjects was high in comparison with the educational attainment of the Spanish population, but this was obviously the result of the necessity of literacy for study inclusion. A survey of people aged 65 or more from the Community of Madrid displayed prevalence of illiteracy of 7.0% and prevalence of more than primary education of 20.7% (vs. 48.6% in the present study) (Morales et al., 2004). Then, results derived from the VP might not be generalizable to people in the low educational strata. Figures regarding prevalence of VRF displayed also some discrepancy, when compared to previous Spanish surveys (Del Barrio et al., 2007).

Analysis of the *APOE* gene showed a prevalence of 17.9% for *APOE* ε4 allele and a mild descent with increasing age, which is similar to other previously studied Spanish populations (Calero et al., 2009) and also consistent with findings from other countries (Corrada et al., 2013).

A prevalence of MCI of 7% was obtained, which falls within the range of 3–19% prevalence reported in previous community-based studies (Busse et al., 2006; Gauthier et al., 2006; Bermejo-Pareja et al., 2008b; Ravaglia et al., 2008; Petersen et al., 2010). Interestingly, virtually, all the cases of MCI presented memory impairment, in contrast with balanced (Busse et al., 2006; Ravaglia et al., 2008) or only mildly disbalanced (Manly et al., 2005; Petersen et al., 2010) prevalence of aMCI and naMCI in previous studies. Our clear predominance of aMCI might be due to selection bias related with good health status (thus lowering the possibility of vascular naMCI) or to methodological issues (underrepresentation or less sensibility of non-memory tests in the baseline assessment). Nevertheless, the predominance of aMCI may be considered positive for the objectives of the VP, since high risk of future AD is expected in MCI when memory is impaired (Petersen et al., 2009).

Determination of AD biomarkers in the CSF was rejected by the participants of the VP. This is in contrast with previous investigations with very high success of CSF consecution (Weiner et al., 2010). This was certainly due to the optional nature of CSF study, but also old age, cultural aspects, or difficult logistics (patients who were interested in the study of CSF biomarkers were referred to a partner hospital) could have contributed. By contrast, blood extraction was fully accepted by the participants and blood samples were successfully collected in virtually all the subjects (99.9%). In addition, the achievement of multi-modal MRI study was high (89.9%). These blood and MRI materials, which are expected to be also longitudinally collected, along with the concomitant social, clinical, and neuropsychological data of the VP, should provide insight in the physiopathological underpinnings of AD and should help to accurately detect the subjects at risk and to provide new avenues for the prevention of this complex and burdensome disease.

## Conflict of Interest Statement

The authors declare that the research was conducted in the absence of any commercial or financial relationships that could be construed as a potential conflict of interest.
